# The “COVID-19 Pandemic Gap” and Its Influence on Oncologic Outcomes of Bladder Cancer

**DOI:** 10.3390/cancers13081754

**Published:** 2021-04-07

**Authors:** Gennadi Tulchiner, Nina Staudacher, Josef Fritz, Christian Radmayr, Zoran Culig, Wolfgang Horninger, Renate Pichler

**Affiliations:** 1 Department of Urology, Medical University Innsbruck, Anichstrasse 35, 6020 Innsbruck, Austria; Gennadi.Tulchiner@i-med.ac.at (G.T.); Nina.Staudacher@i-med.ac.at (N.S.); Christian.Radmayr@i-med.ac.at (C.R.); Zoran.Culig@i-med.ac.at (Z.C.); Wolfgang.Horninger@i-med.ac.at (W.H.); 2 Department of Medical Statistics, Informatics and Health Economics, Medical University of Innsbruck, Schoepfstraße 41, 6020 Innsbruck, Austria; Josef.Fritz@i-med.ac.at

**Keywords:** bladder cancer, urothelial cancer, COVID-19, pandemic, lockdown, diagnosis, staging, outcome, histology

## Abstract

**Simple Summary:**

The COVID-19 pandemic has had a major impact on the entire healthcare system, resulting in severe restrictions of nonemergency clinical services, as well as in the clinical practice of uro-oncology. We performed a retrospective analysis to evaluate outcomes of the COVID-19 pandemic resulting from delayed diagnosis, staging, and treatment of bladder cancer. We showed that the COVID-19 pandemic led to a deferred oncological diagnosis and treatment of bladder cancer. More attention is required to avoid adverse outcomes, with increased rates of advanced and aggressive tumors in patients with primary bladder cancer. Moreover, timely treatment is compulsory in those patients.

**Abstract:**

Coronavirus-19 (COVID-19)-induced effects on deferred diagnosis and treatment of bladder cancer (BC) patients are currently not clarified. The aim of this study was to evaluate outcomes of the COVID-19 pandemic by considering its effects on tumor stage and grade, and to create feasible clinical triage decisions. A retrospective single-center analysis of all patients who underwent diagnostic and surgical procedures due to BC, during January 2019 and December 2020, was performed. Due to COVID-19 lockdowns, significantly fewer (diagnostic and therapeutic) endoscopic procedures were performed in the first 6 months of 2020 compared to 2019 (*p* = 0.002). In patients with a primary diagnosis of BC, a significant increase of high-grade tumors (*p* < 0.001), as well as advanced tumor stages (*p* = 0.014), were noticed during 2020 in comparison to 2019. On the contrary, patients with recurrent BC undergoing risk-adapted surveillance, depending on previous tumor histology, showed no adverse outcomes regarding tumor stage and grade when comparing the pre COVID-19 era with 2020. Thus, more awareness in clinical urologic practice is mandatory to avoid adverse consequences, with increased rates of advanced and aggressive tumors in patients with primary BC. In recurrent BC, an individual risk stratification in order to avoid worse outcomes during the COVID-19 pandemic seems to be justified.

## 1. Introduction

The coronavirus-19 (COVID-19) pandemic has had a major impact on the entire healthcare system around the world [1]. Because of the high risk of virus transmission, no curative therapies, or limited access to vaccines, the primary focus is based on physical distancing. Among other measures, medical facilities were forced to postpone elective surgical and medical procedures to increase hospital capacity [2]. In addition to significant psychological burdens and distress in up to 30% of patients linked with a cancer diagnosis, the pandemic has dramatically changed the care of oncologic patients [3]. Thus, the oncological community faces new, unexpected challenges during the COVID-19 pandemic. A recently published study showed that cancer patients who acquired COVID-19 have a higher risk of significantly worse outcomes, including intensive care unit admission or invasive ventilation [4]. Moreover, the incidence of serious adverse events in asymptomatic COVID-19 positive cancer patients undergoing immunotherapy or chemotherapy was significantly higher compared with COVID-19 negative patients [5]. However, there was no evidence that cancer patients on chemotherapy or other anticancer treatments are at an increased risk of mortality from COVID-19 compared with cancer patients without active treatment [6]. On the contrary, a higher mortality risk for asymptomatic COVID-19 positive patients is evidenced, following elective surgical interventions [7].

The COVID-19 pandemic has had a significant negative impact on cancer diagnosis, with a significant decrease of new cancer diagnoses per general practice, as well as in specialized practices [8]. Thus, a significant reduction in surgery, as well as in access for oncologic therapies, is the logical consequence of the COVID-19 pandemic [9,10]. Focusing on urologic surgeries, a 67% reduction in oncological procedures could be noticed [9]. However, in some cancer entities, delayed surgery leads to a potential increased risk for cancer progression, and needs immediate diagnosis, staging and further treatment [11]. The risks arising from delayed diagnosis and treatment also vary between the different genitourinary tumors, whereby some require a faster intervention than others, including high-grade bladder cancer, advanced renal cell carcinoma, testicular cancer, and penile carcinoma [11]. Thus, uro-oncological conditions with the greatest malignant potential should be prioritized over less aggressive cancers, confirming the highest priority scores for radical orchiectomy/penectomy, transurethral resection of the bladder (TURB) and radical cystectomy (RC) by a survey analysis [12]. However, the effect of medical lockdown on outcomes for underdiagnosed or untreated patients is still unknown. As urothelial cancer tends towards a rapid progression, it is important to prioritize the timely care of those patients. Consequently, our special focus is on patients with this specific tumor entity. In Austria, a decline of the age-standardized incidence rate of bladder cancer (BC) for men (−26%) and women (–28%) was recorded during the last 10 years. During this period, the age-standardized mortality rate was virtually unchanged [13].

A recent review about the risks of delayed treatment for genitourinary cancers provided first recommendations about clinical cancer management during the COVID-19 pandemic [14]. Due to this recommendation, low-grade non-muscle invasive bladder cancers (NMIBC), including low and intermediate European Organization for Research and Treatment of Cancer (EORTC) risk tumors [15], are unlikely to progress from a 3–6 month delay of cystoscopic surveillance and/or transurethral resection of the bladder (TURB) [16]. In contrast, in higher stage (pT1) or higher risk disease, a re-TURB, as well as intravesical bacillus Calmette-Guérin (BCG) therapy, should not be postponed [17,18,19]. Patients with muscle invasive cancers (MIBC) are at higher risk of progression, with a radical cystectomy (RC) delay of more than 12 weeks after diagnosis or completion of neoadjuvant chemotherapy [20,21]. For patients with upper tract urothelial cancer (UTUC), the risk for progression depends on tumor staging and grading. Whereas patients with low-grade UTUC are barely affected [22], a worse pathologic outcome can be expected in patients with a high-grade disease [23]. However, there may be no significant changes regarding survival [23,24].

The aim of this study was to evaluate, for the first time, the possible adverse oncologic outcomes of the COVID-19 pandemic, resulting from a delayed diagnosis, staging and treatment of primary and recurrent urothelial cancers of the bladder, to guide adequate triage decisions for clinicians in the future.

## 2. Patients and Methods

### 2.1. Study Populations and Data Collection

In this retrospective study, all patients who underwent diagnostic procedures (flexible and rigid cystoscopies) for urothelial cancer, and were treated either with TURB and RC from January 2019 until December 2020 at our high-volume center, fulfilled the inclusion criteria. Patients with bladder cancer were stratified into two groups: Primary bladder cancer included patients with a diagnosis during 2019 and 2020, at our department, without history of bladder cancer. As an institutional practice, we prioritized surgery for BC (TURB and RC) being in line with (inter)national urology guidelines for the management of urological conditions during the COVID-19 pandemic [25,26]. Recurrent bladder cancer was defined as tumors with a history of NMIBC undergoing routine surveillance at our institution, according to the European Asscociation of Urology (EAU) guidelines [14]. However, during COVID-19 lockdowns, the frequency of follow-up cystoscopy in recurrent bladder cancer patients was risk-adapted, depending on tumor staging and grading. Follow-up cystoscopies of NMIBC patients with low-risk (primary, solitary, <3 cm, no carcinoma in situ, TaG1, PUNLMP low grade) and intermediate EORTC risk (all tumors between the category of low- and high-risk) were postponed until the end of lockdown. On the contrary, all patients with high-risk NMIBC (T1, G3, high grade, carcinoma in situ and multiple, recurrent and >3 cm TaG1G2/low grade) and/or ongoing one year (intermediate-risk) or three year (high-risk) BCG maintenance (3-weekly instillations 3, 6 and 12 for intermediate-risk; 3, 6, 12, 18, 24, 30 and 36 for high-risk) underwent regular cystoscopic controls, despite reduced medical capacity [14]. Medical records of our single-center bladder cancer database, including demographic and histopathologic data, were collected from patients’ electronic medical records after approval from the local ethics committee (study number: 1006/2017). All tumor specimens after TURB were reviewed in regard of diagnosis, tumor grade (WHO 1973 and 2004/2016) and stage (TNM 2017) by one uropathologist with long-standing experience (I.P.).

### 2.2. Statistical Analysis

Baseline patient characteristics were compared between 2019 and 2020 using Mann–Whitney U tests for continuous variables, and Fisher’s exact tests for categorial variables. Differences in count data (endoscopic diagnostic procedures, cancer cases) between 2019 and 2020 (overall, per half year, and per month) were assessed using exact Poisson tests (also known as c-tests) [27]. Distributions of tumor staging and tumor grading between 2019 and 2020 were compared using Fisher’s exact tests. All statistical tests were two-sided, and statistical significance was defined as *p* < 0.05. Statistical analyses were carried out using GraphPad Prism, version 8.4.1, and R, version 4.0.3. Graphs and figures were generated in GraphPad Prism, version 8.4.1.

## 3. Results

**Figure 1:**
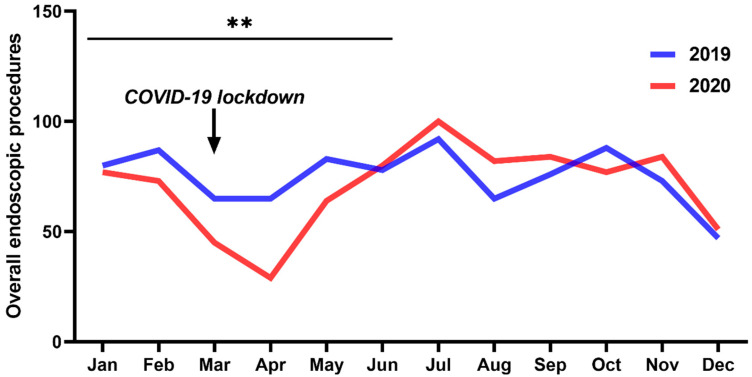
Overall frequency of diagnostic and therapeutic bladder cancer procedures performed during 2020, compared with 2019. ** *p* < 0.01.

Baseline and histopathological characteristics of surgically treated patients with urothelial cancer of the bladder, stratified by intervention, are presented in Table 1. In total, 899 endoscopic procedures (659 simple rigid or flexible cystoscopies without anesthesia and 240 cystoscopies with bladder biopsy or TURBs) were performed at our institution in 2019, compared with 846 endoscopic procedures (637 simple rigid or flexible cystoscopies without anesthesia and 209 cystoscopies with bladder biopsy or TURBs) in 2020, respectively (Figure 1). In detail, the overall frequency of diagnostic and therapeutic procedures for bladder cancer was significantly decreased during the first 6 months of 2020 compared to 2019 (*p* = 0.002) (Figure 1). Interestingly, we noticed a gap between February and June 2020, with a maximum drop in March and April 2020 (*p <* 0.001) due to the specific COVID-19 lockdown measures in Austria. However, after this COVID-19 pandemic gap, a steep rise in diagnostic procedures could be registered during the second half of 2020, so that the total number of diagnostic procedures over the whole year did not significantly differ between 2019 and 2020 (*p* = 0.213).

**Figure 2:**
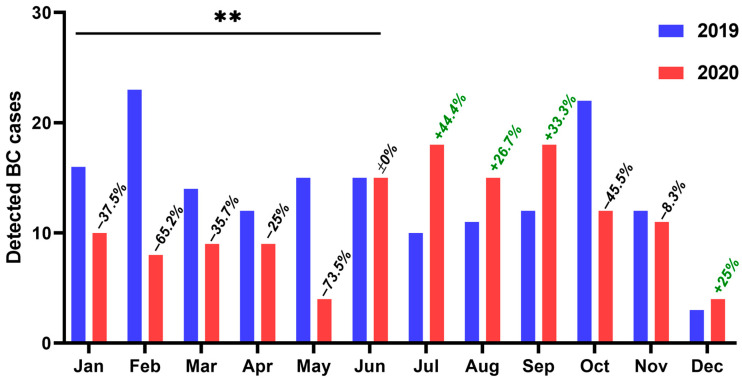
Total number of diagnosed bladder cancer cases in 2020, compared with 2019. ** *p* < 0.01.

Focusing on bladder cancer diagnosis, we confirmed overall less diagnosed BCs in the first half of 2020 compared to 2019 (*p* = 0.001) (Figure 2). In detail, a decline in newly diagnosed BC was noticed during the lockdown in March 2020, but, in addition, already in February 2020 (−65.2%; *p* = 0.011) as well as in May 2020 (−73.5%; *p* = 0.019), a significant reduction in tumor diagnosis became obvious (Figure 2). However, a strong increase in new BC diagnoses could be recorded after the first lockdown in Austria, already starting in July 2020 compared to 2019 (+44.4%, *p* = 0.185). Due to this fact, the total number of bladder cancer diagnoses for the whole year was outbalanced, without any significant differences, comparing between 2020 and 2019 (*p* = 0.072).

**Figure 3:**
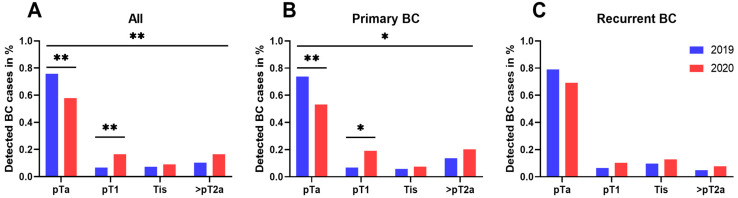
Tumor staging compared between 2020 and 2019. (**A**) total BC population (*n*[2019] = 165; *n*[2020] = 133), (**B**) patients with primary bladder cancer (*n*[2019] = 103; *n*[2020] = 94), and (**C**) patients with recurrent BC during oncological follow-up (*n*[2019] = 62; *n*[2020] = 39). * *p* < 0.05; ** *p* < 0.01.

Regarding the oncologic outcomes of all bladder cancer patients, our results confirm an overall significant increase in high-grade tumors (*p* = 0.001) and higher tumor stages (*p* = 0.006) during 2020 compared with 2019, with significantly fewer pTa tumors (*p* = 0.001) but more pT1 tumors (*p* = 0.009) (Figure 3a,b).

The reported differences of tumor stage outcome between 2019 and 2020 can also be observed when stratified by monthly diagnosis, with more pTa tumors during the first 6 months in 2019 (*p* ≤ 0.001) and more pT1 tumors during the last 6 months in 2020 (*p* = 0.003) (Supplementary Figure S1).

**Figure 4:**
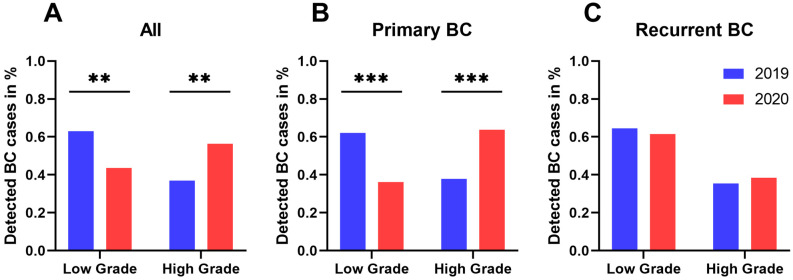
Tumor grading compared between 2020 and 2019. (**A**) total BC population (*n*[2019] = 165; *n*[2020] = 133), (**B**) patients with primary BC (*n*[2019] = 103; *n*[2020] = 94), and (**C**) patients with recurrent BC during oncological follow-up (*n*[2019] = 62; *n*[2020] = 39). ** *p* < 0.01; *** *p* < 0.001.

**Figure 5:**
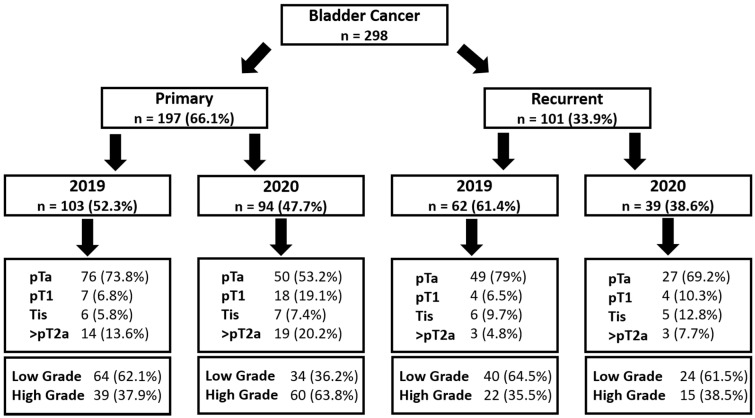
All diagnosed cancer cases stratified into primary bladder cancers and recurrent tumors, compared for the years 2019 and 2020.

Focusing on primary BC, the incidence of high-grade tumors was 37.9% for 2019 compared to 63.8% in 2020 (*p* < 0.001), whereas the rate of pTa tumors was significantly decreased during 2020 as compared to 2019 (53.2% vs. 73.8%; *p* = 0.003). Higher tumor stages, such as pT1 (19.1% vs. 6.8%; *p* = 0.010) or ≥pT2 (20.2% vs. 13.6%; *p* = 0.254), were noticed, resulting in overall higher tumor stages (*p* = 0.006) during 2020 (Figure 3b, Figure 4b and Figure 5).

On the contrary, in patients with recurrent BC, no adverse oncologic outcomes concerning tumor staging or grading could be detected when comparing 2019 with 2020 (Figure 3c and Figure 4c). Moreover, no significant differences regarding tumor histology were shown in patients undergoing radical cystectomy (*n* = 44) between 2019 and 2020 (Table 1).

## 4. Discussion

On 12 March 2020, the World Health Organization (WHO) declared COVID-19 a global pandemic. In Austria, the first COVID-19 lockdown was implemented on 15 March 2020, also resulting in severe restrictions affecting the entire healthcare system. To prevent the spread of the virus and to deploy medical staff and capacity towards the management of COVID-19 cases, regular medical services, such as routine examinations and controls, as well as elective, nonurgent surgical interventions, were almost no longer possible. Thus, nonemergency clinical services, such as routine oncological follow-up controls, as well as screening procedures and elective surgeries, were deprioritized by our hospital operator. Moreover, lockdowns and increased fear due to the COVID-19 impact in other countries had a dramatic effect on first presentation and referral of patients to medical institutions. As a result, significantly fewer cancer diagnoses were reported during the COVID-19 pandemic in different countries [28,29].

In line with these findings, significantly fewer diagnostic and therapeutic procedures were performed at our institution during 2020 compared to 2019, due to restrictive health policy, resulting in a decreased BC detection with a diagnostic gap during the first COVID-19 lockdown between March and June 2020. Interestingly, we also already observed a decrease in newly diagnosed BC tumors in February 2020. We hypothesize that the pandemic situation in Italy, Austria’s neighbor country, which was already worse in the beginning of 2020, might have increased the awareness of patients, so they avoided taking advantage of further diagnostic examinations, despite symptoms. Although there was no significant difference in total histologically proven tumors between 2019 and 2020, we are able to show that there was a shift, with a significantly lower cancer detection rates in the first half of 2020. Interestingly, there is a repeated decline of cancer diagnosis after October 2020. A possible explanation could be the increasing COVID-19 incidence and, subsequently, a second wave in Austria, with renewed restrictive measures.

To our best knowledge, we present, for the first time, results about the negative impact of the COVID-19 pandemic on histological outcome of patients with primary BC. Overall, we observed a significant switch to more aggressive (high grade) and advanced tumors (pT1) in 2020 compared to 2019. Moreover, when stratifying patients in primary and recurrent tumors, we were able to demonstrate that the group with newly diagnosed cancer was mainly affected by health policy restrictions, with consecutive adverse oncologic outcomes, in contrast to recurrent bladder cancer patients. This finding could be related to the fact that patients who are in regular oncological surveillance are classified and stratified based on their probabilities of recurrence and progression, according to our institutional practice and following the EAU guidelines [14,15]. Therefore, we were able to reprioritize our recurrent bladder cancer patients undergoing surveillance, favoring the diagnostic and therapeutic procedures for those at higher risk of progression, despite reduced medical resources. In contrast, the follow-up controls of BC patients with low- or intermediate-risk were postponed until the end of lockdown.

On the other hand, primary bladder cancer diagnosis was strongly restricted because of low accessibility to healthcare services (clinicians, as well as urologists in private practice) being a possible reason for their advanced disease at first diagnosis. Moreover, patients probably avoided routine medical check-ups because of the fear of COVID-19 exposure. Consequently, an increase in undetected, or delayed, tumor diagnoses, leading to a significant tumor upstaging and upgrading, was recorded. Moreover, BC is highly heterogeneous at both the histologic and molecular level [30]. Multiple molecular pathways are linked with bladder carcinogenesis [31] and various genetic alterations are associated with progression [32,33,34], recurrence [32], or BCG therapy response [35]. Thus, early diagnostic and therapeutic interventions in primary bladder cancer are essential for an accurate histopathological and molecular staging to enable a more personalized treatment approach, and to achieve the best oncologic outcome.

Our study certainly has its limitations. First of all, it is a retrospective analysis comprising the potential for selection bias. Because of the lack of medical records, we were unable discover whether patients already had symptoms, such as hematuria, during the lockdown in 2020, resulting in a time delay from initial symptoms to the first urologic visit, as well as a delay until surgical intervention. Furthermore, our data represent the situation from only one center in that unprecedented and unpredictable time. On the other hand, hospitals follow their own internal guidelines and restrictions during the COVID-19 pandemic, leading to further confounders that could impede the interpretation of data. Finally, a larger population with a longer follow-up is mandatory to prove whether such a pandemic has an additional negative impact on survival outcomes, especially in locally advanced tumors or MIBC.

## 5. Conclusions

In conclusion, in BC patients undergoing routine surveillance, our data support that a balanced and risk-adapted decision process, depending on previous tumor histology, is sufficient for the optimal management of oncologic follow-up during the COVID-19 pandemic, without worsening prognosis in those patients [14]. In detail, it is safe to defer cystoscopic surveillance for recurrence in patients with known low- and intermediate-EORTC risk NMIBC during the COVID-19 pandemic. On the contrary, we urge caution and highlight the importance of cystoscopic surveillance, specifically in patients with high-risk NMIBC and all (intermediate and high-risk) patients undergoing BCG maintenance.

Adverse histopathological outcomes during the COVID-19 pandemic for patients with primary diagnosis of bladder cancer, resulting in an increased incidence of advanced and more aggressive tumor stages, could be observed. Thus, the increasing oncological risk of delayed diagnosis in patients with newly diagnosed bladder cancer demands more awareness in clinical practice. Earlier surgical interventions, irrespective of the risk of a potential COVID-19 exposure, with an accurate histopathological staging, should be considered in the management of primary bladder cancer patients.

## Figures and Tables

**Table 1:** Baseline patient characteristics.

		2019	2020	*p* Value
**Endoscopic Surgery**	**Patients No.**	**240**	**209**	
	**Age**			0.381 †
	Mean ± SD/Median (IQR)	70.2 ± 12.6/72 (25–95)	71.7 ±10.6/72 (38–92)	
	**Sex**			1.000 *
	W (%)/M (%)	39 (16.3)/201 (83.7)	34 (16.3)/175 (83.7)	
	**Histology**			0.008 *
	No malignancy (%)	75 (31.3)	76 (36.4)	
	pTa (%)	125 (52.1)	77 (36.8)	
	pT1 (%)	11 (4.6)	22 (10.5)	
	CIS (%)	12 (5)	12 (5.7)	
	pT2a (%)	17 (7.1)	22 (10.5)	
	**Grade**			0.002 *
	No malignancy (%)	75 (31.3)	76 (36.4)	
	Low (%)	104 (43.3)	58 (27.8)	
	High (%)	61 (25.4)	75 (35.9)	
**Radical Cystectomy**	**Patients No.**	22	22	
	**Age**			0.647 †
	Mean ± SD/Median (IQR)	67.6 ± 10.8/66.5 (38–83)	69.4 ± 9.3/70 (46–86)	
	**Sex**			0.162 *
	W (%)/M (%)	8 (36.4)/14 (63.6)	3 (13.6)/19 (86.4)	
	**Histology**			0.762 *
	pT1	1 (4.5)	2 (9.1)	
	CIS	1 (4.5)	-	
	pT2	2 (9.1)	5 (22.7)	
	pT3	5 (22.7)	4 (18.2)	
	pT4	2 (9.1)	2 (9.1)	
	ypT0	6 (27.3)	5 (22.7)	
	ypT1	1 (4.5)	3 (13.6)	
	ypTis	1 (4.5)	1 (4.5)	
	ypT2	1 (4.5)	-	
	ypT3	2 (9.1)	-	
	**NAC**			0.763 *
	Yes (%)/No (%)	11 (50)/11 (50)	9 (40.5)/13 (59.1)	
	**Delay > 3 Months**			1.000 *
	Yes (%)/No (%)	1 (4.5)/21 (95.5)	2 (9.1)/20 (90.5)	

* Fisher’s exact test; † Mann–Whitney U-test; NAC = neoadjuvant chemotherapy.

## Data Availability

The data presented in this study are available on request from the corresponding author.

## Supplementary Materials

The following are available online at https://www.mdpi.com/article/10.3390/cancers13081754/s1, Figure S1: Differences of tumor stage between 2019 and 2020 stratified by monthly diagnosis.

## Abbreviations

BC	bladder cancer
BCG	bacillus Calmette-Guérin
COVID-19	coronavirus-19
EAU	European Asscociation of Urology
EORTC	European Organization for Research and Treatment of Cancer
NMIBC	nonmuscle invasive bladder cancers
MIBC	muscle invasive cancers
RC	radical cystectomy
TURB	transurethral resection of the bladder
UTUC	upper tract urothelial cancer
WHO	World Health Organization
