# Supplementary material for: The “COVID-19 Pandemic Gap” and Its Influence on Oncologic Outcomes of Bladder Cancer
Source: Cancers (Basel). 2021 Apr 7;13(8):1754. doi: 10.3390/cancers13081754 (PMC8067623; doi:10.3390/cancers13081754)
Supplement: Supplementary file 1 [file cancers-13-01754-s001.pdf]

cancers

MDPI

Supplementary materials: The "COVID-19 Pandemic Gap" and Its Influence on Oncologic Outcomes of Bladder Cancer

Gennadi Tulchiner, Nina Staudacher, Josef Fritz, Christian Radmayr, Zoran Culig, Wolfgang Horninger and Renate Pichler

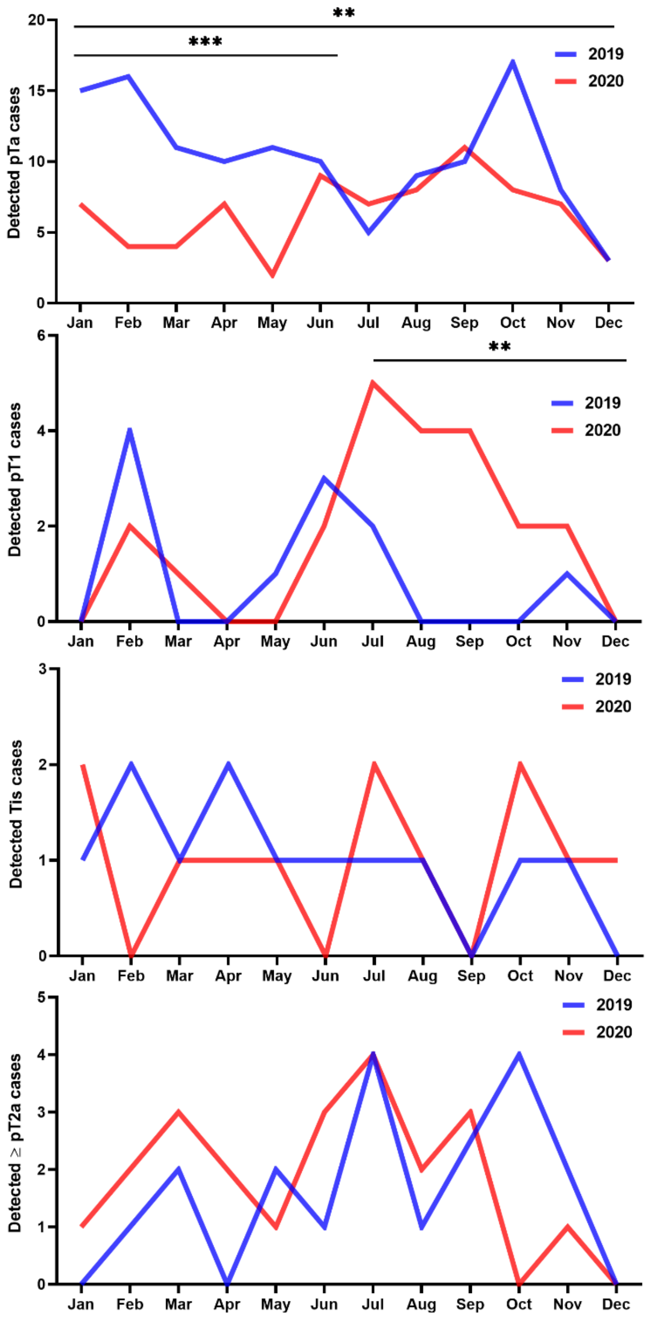

Figure S1. Differences of tumor stage between 2019 and 2020 stratified by monthly diagnosis.
